# Investigating the Kidney–Gut–Brain Axis in CKD: Uremic Toxins and Brain Microhemorrhages

**DOI:** 10.3390/ijms27136020

**Published:** 2026-07-04

**Authors:** Yitong Zhao, Su Mi Lee, Whitney Li, David Floriolli, Peter Chang, Yoko Narasaki, Amy S. You, Kamyar Kalantar-Zadeh, Connie M. Rhee, Han Liu, Tiffany Tran, Annlia Paganini-Hill, Mark Fisher, Wei Ling Lau

**Affiliations:** 1 Division of Nephrology, Department of Medicine, University of California, Irvine, CA 92697, USA; 2 Department of Internal Medicine, College of Medicine, Dong-A University, Busan 49201, Republic of Korea; 3 Department of Radiology, University of California, Irvine, CA 92697, USA; 4 Division of Nephrology, David Geffen School of Medicine, University of California Los Angeles, Los Angeles, CA 90073, USA; 5 The Lundquist Institute for Biomedical Innovation at Harbor-UCLA Medical Center, Torrance, CA 90502, USA; 6 Beckman Laser Institute and Medical Clinic, University of California, Irvine, CA 92697, USA; 7 Department of Neurology, University of California, Irvine, CA 92697, USA; 8 Department of Pathology & Laboratory Medicine, University of California, Irvine, CA 92697, USA; 9 Department of Physiology & Biophysics, University of California, Irvine, CA 92697, USA

**Keywords:** chronic kidney disease, gut microbiome, uremic toxins, behavior testing, brain microbleeds

## Abstract

Alterations of gut microbiota are common in chronic kidney disease (CKD) and contribute to increased uremic toxins including indoxyl sulfate (IS), p-cresyl sulfate (pCS) and trimethylamine N-oxide (TMAO), which are linked to cerebrovascular disease risk. This study examined the kidney–gut–brain axis in CKD mice and in dialysis patients. Male and female mice with adenine-induced CKD were fed a high-amino-acid (HAA) diet to increase precursors of gut-derived uremic toxins. A subgroup of mice received antibiotics in drinking water to suppress gut microbiota and evaluate its role in toxin generation. Behavior tests, gut microbiome composition and brain histology for cerebral microhemorrhages were analyzed. CKD mice had higher serum levels of creatinine, cystatin C and gut-derived toxins, a 2.5-fold increase in brain microhemorrhages, and decreased locomotor activity. The HAA diet significantly increased serum TMAO but not IS and pCS, and all three toxins were reduced by antibiotic therapy. Sex differences were observed; in male animals, higher TMAO was associated with increased brain microhemorrhages, whereas in female mice, pCS was associated with brain microhemorrhage burden. The suppression of toxins with antibiotics improved working memory in male animals. Gut microbiota analysis revealed the expansion of Lactobacillus and Ileibacterium in CKD mice. The HAA diet and antibiotics altered gut microbiota composition without changing alpha diversity. The human study utilized biobanked serum samples and a retrospective review of brain imaging scans in a hemodialysis patient cohort; TMAO levels were associated with increased lobar microbleeds. Our study supports a role for bacterial-derived uremic toxins in the kidney–gut–brain axis and cerebral microhemorrhage formation in CKD.

## 1. Introduction

In chronic kidney disease (CKD), toxins derived from altered gut microbial metabolism enter the systemic circulation and promote inflammation and vascular injury [1,2,3,4]. Major gut-derived toxins such as indoxyl sulfate, p-cresyl sulfate and trimethylamine N-oxide (TMAO) have been shown to induce endothelial dysfunction in vitro and are associated with cognitive impairment and increased mortality in CKD patients [5,6,7,8]. These toxins predominantly arise from amino acid catabolism. Indoxyl sulfate results from the metabolism of tryptophan into indole by intestinal bacteria, which is then sulfated in the liver [9,10]. P-cresol is generated from phenylalanine and tyrosine and is conjugated to form the circulating toxin p-cresyl sulfate [11,12]. TMAO is derived from the bacterial metabolism of quaternary amines such as phosphatidylcholine, betaine, or L-carnitine to release trimethylamine, which is converted to TMAO by flavin monooxygenase enzymes in the liver [1,13].

An emerging area of study is the kidney–gut–brain axis, i.e., how gut dysbiosis in the uremic milieu contributes to cerebrovascular disease. Cognitive impairment is more prevalent in persons with advanced CKD than in the general population and affects 30–70% of the end-stage kidney disease (ESKD) population [14,15,16]. Cerebral small vessel disease is the major precursor to cognitive decline in CKD [17,18,19,20] and includes the neuropathologic entity cerebral microhemorrhages, which may manifest as microbleeds ≤ 10 mm on magnetic resonance imaging (MRI). The prevalence of microbleeds increases from 14% in CKD stage 3 to 34% in CKD stage 5 [21], to ~50% in ESKD patients [22,23,24,25].

Our group previously reported a 2–2.5-fold increase in brain microhemorrhages with impairment of the blood–brain barrier in two mouse CKD models [26]. In the current study, we examined the relationship between dietary precursors of gut-derived uremic toxins and microhemorrhage formation in the mouse adenine CKD model. Further, we utilized broad-spectrum antibiotics to clarify the central role of the gut microbiota in generating circulating levels of indoxyl sulfate, p-cresyl sulfate and TMAO. Finally, we analyzed associations between blood toxin levels and brain microbleeds in a retrospective human database of hemodialysis patients. Our overarching hypothesis was that uremic toxins contribute to manifestations of cerebral small vessel disease.

## 2. Results

### 2.1. Mouse Study

Table 1 summarizes aggregate female and male data in the six mouse experimental groups. CKD animals on an HAA diet with/without Abx treatment showed a significant decrease in serum creatinine levels compared with CKD mice not on the HAA diet. Data stratified by sex are shown in Supplemental Table S1. Serum creatinine and cystatin C were higher in male vs. female CKD animals.

#### 2.1.1. Gut-Derived Uremic Toxins

**Figure 1:**
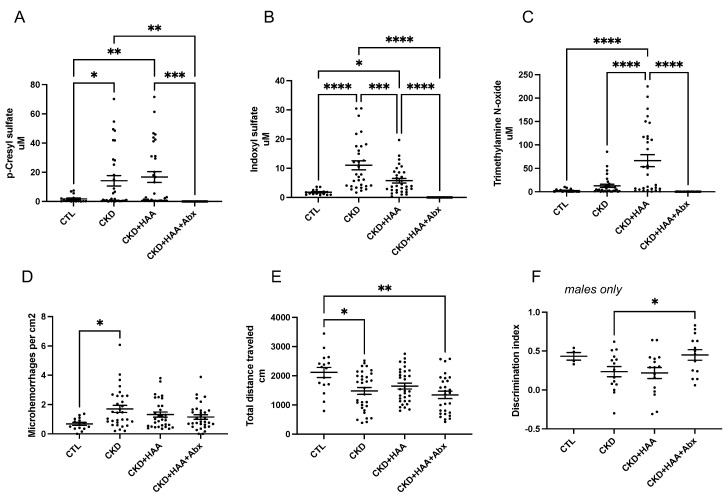
Altered serum uremic toxin levels, brain hemorrhages, and behavior scores in CKD animals (combined male and female data). Serum levels of (**A**) p-cresyl sulfate, (**B**) indoxyl sulfate, and (**C**) trimethylamine N-oxide at study termination in control (CTL) and chronic kidney disease (CKD) animals. (**D**) Microhemorrhage counts normalized to total brain surface area. (**E**) Distance traveled in Open Field Test. (**F**) Discrimination index scores in male mice. Abx = antibiotics in drinking water; HAA = high-amino-acid diet. Data points represent individual animals with the group means indicated by the horizontal lines; for each sex *n* = 8 for control and *n* = 16 for each CKD experimental group (total n of 16 and 32 respectively per CTL and CKD group); ANOVA with post hoc Holm–Sidak test; * *p* < 0.05, ** *p* < 0.01, *** *p* < 0.001, **** *p* < 0.0001.

In the CKD model, levels of indoxyl sulfate and p-cresyl sulfate were increased; these levels were substantially lowered with antibiotic treatment. Levels of TMAO increased significantly with CKD combined with the HAA diet, and these levels were also reduced with antibiotic treatment (Table 1, Figure 1). When analyzed by sex, the gut-derived toxins p-cresyl sulfate and indoxyl sulfate were generally higher in male mice than in female animals, whereas TMAO was higher in females (Supplemental Table S1).

#### 2.1.2. Behavior Testing

Aggregate male and female data demonstrated decreased locomotor activity in CKD animals, and this was not changed with HAA or HAA + Abx treatment (Table 2 and Figure 1E). With Novel Object Recognition testing, male CKD + HAA + Abx animals demonstrated improved discrimination index (DI, recognition memory) scores compared with untreated CKD male mice (Figure 1F); this effect was not observed in female mice.

#### 2.1.3. Brain Microhemorrhages

**Figure 2:**
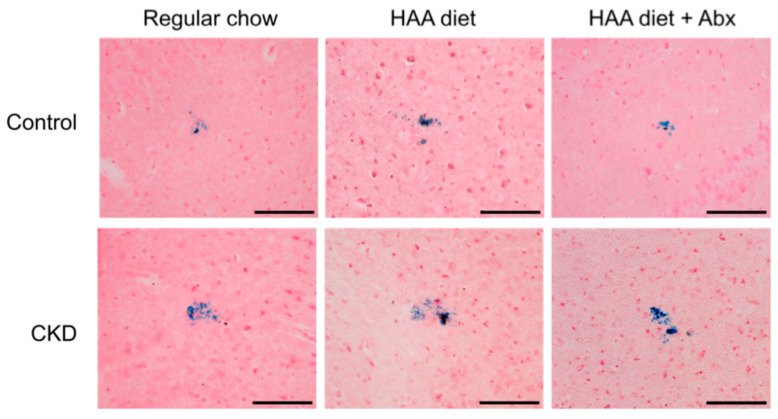
Representative images of mouse brain microhemorrhages on Prussian blue histology: 20× objective, scale bar = 100 μm. Abx = antibiotics in drinking water; CKD = chronic kidney disease; HAA = high-amino-acid diet.

Brain microhemorrhage data are summarized in Table 1 and Figure 1, and representative figures are shown in Figure 2. Microhemorrhage counts increased 2.5-fold in CKD animals compared with controls (CTL) (Figure 1D); counts in CKD animals in the HAA and HAA + Abx groups remained higher than the CTL, but these increases were not statistically significant. When analyzed by sex, female CKD mice had significantly increased microhemorrhage counts compared with female CTL mice (*p* < 0.05) and this difference approached statistical significance in male mice (*p* = 0.06) (Supplemental Table S1). In male mice, microhemorrhage counts trended lower in CKD + HAA + Abx compared with untreated CKD animals (*p* = 0.06).

#### 2.1.4. Gut Microbial Diversity

**Figure 3:**
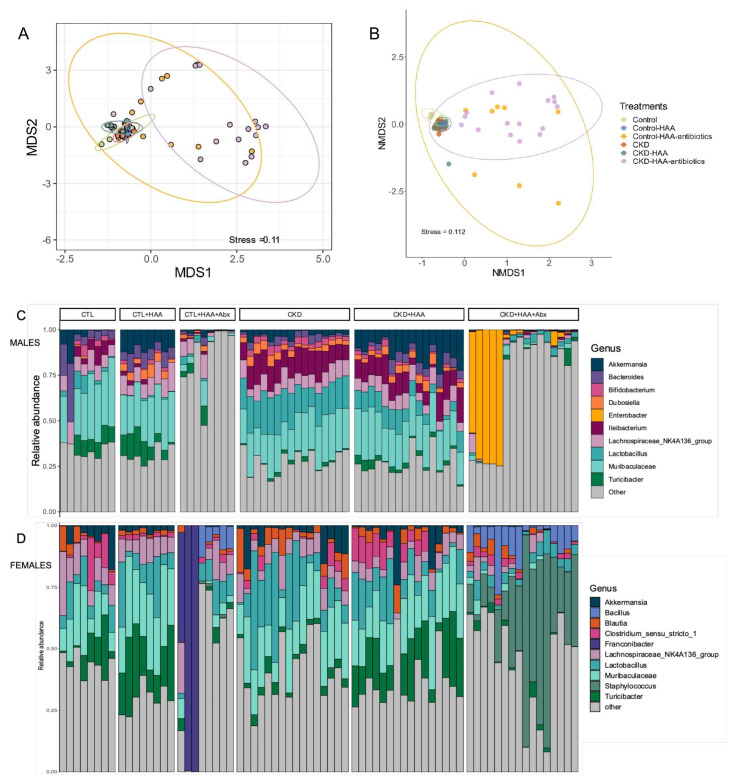
Impact of HAA and antibiotic changes on the gut microbiome in mice. Non-metric multidimensional scaling (NMDS) plot of beta diversity (difference in microbial community composition between samples) demonstrated wider dispersion of samples with antibiotic therapy in male (**A**) and female (**B**) mice. Taxa bar plot of relative abundance of gut bacterium genera in male (**C**) and female (**D**) mice demonstrated sex differences with antibiotic therapy. Abx = antibiotic cocktail in drinking water, CKD = chronic kidney disease mice, CTL = control mice, HAA = high-amino-acid diet.

Adenine CKD animals demonstrated an expansion of *Lactobacillus* and *Ileibacterium* genera compared with CTL mice. Abx treatment decreased the abundance of microbial taxa in CTL and CKD animals (Shannon diversity index trended lower, Table 1) and the microbiome composition of Abx-treated animals clustered more apart from the other groups (Figure 3A,B).

Overall, male mice had a lower Shannon diversity index than female animals. PERMANOVA testing demonstrated that cage (housing) effects accounted for 43% of the community variance among the male experimental groups (*p* < 0.001). Abx treatment, CKD and HAA diet respectively accounted for 23%, 11% and 6% of the microbial community variance (*p* < 0.001 for each factor) in males, and 33%, 3% and 10% respectively (*p* = 0.001 for each factor) in female mice. The relative abundance of taxa in male and female mice was calculated using the rarefied OTU table and the top 10 genera are shown in Figure 3C,D. In male mice, the HAA diet increased the relative abundance of *Akkermansia* in both CTL and CKD mice. Abx treatment decreased the abundance of microbial taxa in CTL and CKD animals. However, in about one-third of male CKD + HAA + Abx mice, a marked increase in *Enterobacter* species was observed. In female mice, the HAA diet increased the relative abundance of *Turicibacter* in both CTL and CKD mice. Although Abx treatment decreased the abundance of microbial taxa in CTL and CKD animals, *Franconibacter* and *Staphylococcus* species were increased in female CTL + HAA + Abx and CKD + HAA + Abx animals, respectively.

#### 2.1.5. Correlation Analysis with Uremic Toxins

**Figure 4:**
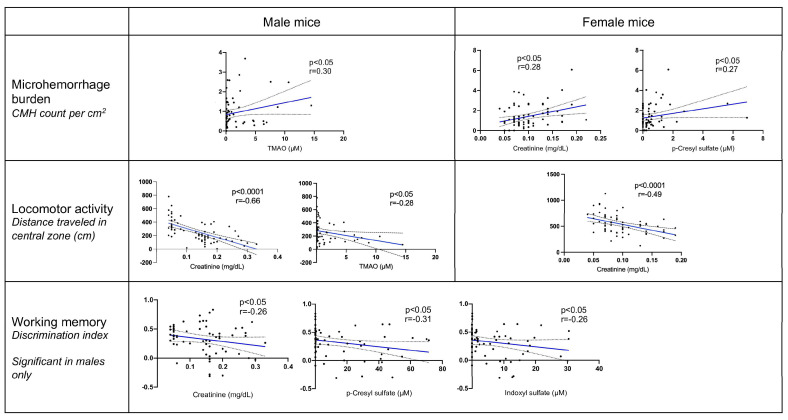
Correlation analyses between uremic toxins with behavior outcomes and brain microhemorrhage burden in control and chronic kidney disease mice, stratified by sex. CMH = cerebral microhemorrhages. Data points represent individual mice; Spearman’s correlation coefficient (r) with 95% confidence interval; *n* = 72 each for males and females.

In the overall cohort, creatinine and gut-derived toxins correlated with altered locomotor activity (Supplemental Table S2). Analysis by sex was performed to determine correlations between uremic toxin levels, brain microhemorrhages and behavior testing scores. Serum creatinine demonstrated a significant positive correlation with the three gut-derived uremic toxins and was associated with decreased locomotor scores on the Open Field Test (Figure 4; Supplemental Table S2). In male mice, TMAO correlated with higher brain microhemorrhage count and decreased locomotor activity. Higher creatinine, p-cresyl sulfate and indoxyl sulfate levels were correlated with decreased working memory (lower DI scores) in male animals; this was not observed in females. In female mice, creatinine and p-cresyl sulfate were positively correlated with microhemorrhage counts. Multivariable regression models of each gut-derived toxin adjusting for creatinine with/without the other gut-derived toxins (Supplemental Table S3) confirmed the varying relationships between gut-derived toxins and microhemorrhage burden.

### 2.2. Clinical Cohort

The hemodialysis patient group included 15 males and 15 females, aged 58.5 ± 3.1 years, who had been on dialysis for 54.1 ± 3.1 months at the time of blood sampling (Table 3). Brain MRI was completed within 0.03–20.6 months of blood draw, with a median interval of 2.4 months. Higher TMAO levels were associated with the presence of lobar microbleeds (β = 0.61, *p* = 0.02; odds ratio [OR] = 1.84 with 95% CI [1.10, 3.09]). Mean TMAO was 6.2 ± 0.7 vs. 5.1 ± 0.4 µM in patients with vs. without lobar microbleeds. Indoxyl sulfate was negatively associated with presence of lobar microbleeds (β = −0.48, *p* = 0.02; OR = 0.62 with 95% CI [0.41, 0.92]). Toxin levels did not correlate with infratentorial or deep microbleeds.

## 3. Discussion

Consistent with our prior work [26,27,28], this study again demonstrates that CKD increases brain microhemorrhages and provides new insights into the kidney–gut–brain axis. In the current study, CKD mice demonstrated alterations in the gut microbiome, increased serum levels of gut-derived uremic toxins, and neurobehavioral abnormalities (most notably decreased locomotor activity). Sex differences were observed; in female animals, higher p-cresyl sulfate was associated with increased brain microhemorrhages, whereas in male mice, TMAO was associated with brain microhemorrhage burden. In the human hemodialysis cohort, higher TMAO correlated with lobar microbleeds. The HAA diet increased serum levels of the water-soluble toxin TMAO but did not increase brain microhemorrhages, suggesting that kidney disease severity was the main determinant of microvascular injury. Broad-spectrum Abx therapy effectively suppressed serum levels of gut-derived toxins, confirming the gut microbiota as the major source of these toxins, and was associated with improved working memory in male CKD mice.

The HAA diet was used as the precursor to the toxins of interest and showed significant variability in the resulting serum toxin levels. TMAO was consistently elevated with the HAA diet, whereas indoxyl sulfate and p-cresyl sulfate exhibited more variable changes, likely due to biochemical characteristics. TMAO is a water-soluble metabolite derived from the gut microbial conversion of dietary choline, and its circulating levels are highly responsive to dietary intake. In contrast, indoxyl sulfate and p-cresyl sulfate are protein-bound toxins generated from the microbial fermentation of tryptophan and tyrosine, respectively, and their levels are influenced by more complex regulation including renal clearance, extensive albumin binding, and gut microbial composition [29,30]. Despite lower TMAO levels in male vs. female mice (consistent with our prior report [28]), there was a notable sex difference whereby TMAO correlated with brain microhemorrhage burden and decreased locomotor activity only in males (Figure 4). Our prior in vitro work supports a role for gut-derived toxins in promoting brain endothelial dysfunction in the uremic milieu; incubation with the three major gut-derived uremic toxins combined with urea and lipopolysaccharide significantly disrupted endothelial monolayer integrity [27]. Further mechanistic studies are needed to address the effect of sex on brain microvascular injury, potentially through hormonal regulation and differences in vascular vulnerability.

The lower serum creatinine observed in the CKD + HAA and CKD + HAA + Abx was likely related to hemodynamic adaptations, potentially masking ongoing kidney injury. High protein or amino acid intake influences nitric oxide signaling and tubuloglomerular feedback, increasing renal plasma flow and glomerular filtration rate, thereby enhancing the clearance of creatinine and cystatin C [31,32]. While not the focus of the current study, it is proposed that long-term high dietary protein intake and kidney hyperfiltration perpetuates glomerular injury and proteinuria, leading to CKD progression [33,34]. Within the 5-week diet study period in adenine CKD mice, the HAA diet significantly increased serum water-soluble TMAO but not p-cresyl sulfate and indoxyl sulfate, suggesting that the latter protein-bound toxins are less modified by dietary changes.

**Figure 5:**
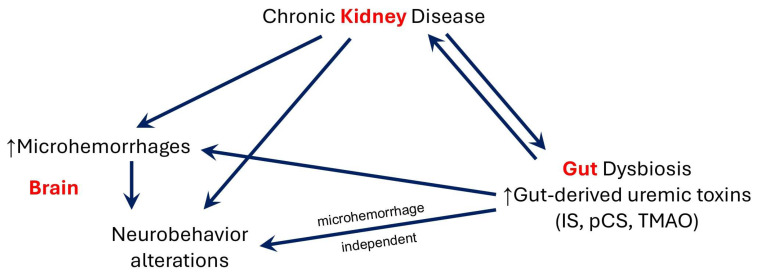
Proposed role of bacterial-derived uremic toxins in the kidney–gut–brain axis in chronic kidney disease (CKD). In our mouse study, degree of CKD was the main predictor of elevated serum levels of gut-derived toxins including indoxyl sulfate (IS), p-cresyl sulfate (pCS) and trimethylamine N-oxide (TMAO). These toxins were associated with brain microhemorrhages and neurobehavior changes.

Within male mice, TMAO was associated with microhemorrhage burden and decreased locomotor activity, whereas p-cresyl sulfate and indoxyl sulfate correlated with impaired working memory (DI scores). The locomotor deficits observed with the adenine CKD model have been previously described [35,36]. In our study, male mice were less active than female mice, and CKD mice showed consistently less activity than sex-matched controls. Serum creatinine (which reflects overall degree of kidney dysfunction) was the most consistent predictor of brain microvascular disease and impaired behavior. Indeed, in a unilateral nephrectomy CKD model, intraperitoneal administration of p-cresol sulfate [37] or indoxyl sulfate [38] promoted anxiety and cognitive impairment behaviors, accompanied by decreased neuron survival and increased neuroinflammation (vascular-independent mechanisms). The observed associations between gut-derived uremic toxins with brain microhemorrhages and DI scores suggest that toxin-induced brain injury may involve both microhemorrhage-dependent and -independent pathways (Figure 5).

Gut microbial analysis demonstrated increased *Lactobacillus* and *Ileibacterium* in CKD animals, and sex-specific changes were noted with HAA and Abx interventions. Microbiome studies are challenging because of the wide variability in microbial communities within CTL and CKD individuals. A recent meta-analysis of rodent CKD models did not identify reproducible patterns of gut dysbiosis and noted that cohort effects accounted for 69% of the total sample variance (*p* < 0.001), while CKD only accounted for 1.9% of the variance [39]. Similarly, in the current study, cage (cohort) effects accounted for about 40% of the community variance among the treatment groups; we noted that CKD accounted for 3–11% of the variance. The Shannon index, a marker of microbial diversity, was higher in female mice than male mice. This sex difference has also been observed in humans, whereby women have higher gut microbial diversity than men [40,41]. Additionally, we noted sex differences in the impact of CKD on gut microbial diversity: 11% of microbial variance was explained by CKD in males vs. 3% in females. In male mice, HAA diet was associated with increased *Akkermansia*, a mucus-degrading bacteria that becomes more abundant in low-fiber-diet or fasting states and has been studied extensively as a potential probiotic with beneficial immunomodulatory properties [42,43,44]. A prior study in 5/6 nephrectomized CKD mice, in which a low-protein diet was supplemented with α-ketoacid, noted a similar increase in *Akkermansia* abundance [45]. The increase in *Enterobacter* in a subgroup of CKD + HAA + Abx animals was independent of cage effects and suggests evolving Abx resistance that will need further study.

In the human retrospective study of hemodialysis patients, we noted a significant correlation between TMAO and lobar microbleeds—similar to the relationship observed in male mice. Observational studies in patients with stroke or transient ischemic attack have noted an association between TMAO and white matter hyperintensities [46,47], but not with microbleeds [46]. However, these studies were not in CKD patients, who have complex metabolic derangements and significantly higher circulating levels of gut-derived toxins. In the current dialysis cohort, there was a paradoxical inverse relationship between indoxyl sulfate and lobar microbleeds; further work with larger sample sizes is needed to verify this finding.

We acknowledge several limitations in the current work. In the mouse study, we did not specifically investigate the effects of dietary adenine on the gut microbiome [48]. Furthermore, the observed effects with Abx therapy cannot be attributed to gut toxin suppression alone as we could not account for Abx effects on systemic inflammation or nutritional status [49,50], which may also modulate brain microvasculature and behavior. Future studies using more specific targeted microbial or metabolic interventions are needed to confirm (a) specific microbes responsible for toxin generation, and (b) cognitive impact attributable to gut-derived toxins. Behavior studies in CKD models should be interpreted cautiously. The principal finding was reduced locomotion with CKD, which may reflect malaise and weight loss in this model. However, we did observe improved memory function (discrimination index) in male mice treated with a HAA diet and antibiotics. This finding is consistent with a specific effect preserving memory, and deserves further study. Major limitations of the human study are the small cohort size, the variable field strength of MRI studies, and the varying time intervals between toxin measurements and MRI. As both uremic toxin levels and microhemorrhage burden may change over time, the associations need to be interpreted cautiously and the current findings are deemed exploratory. Adjustment for comorbidities in our human study (hypertension, diabetes mellitus, stroke history) was not feasible due to the small sample size.

## 4. Materials and Methods

### 4.1. Mouse Study

#### 4.1.1. Adenine CKD Model and Controls

**Figure 6:**
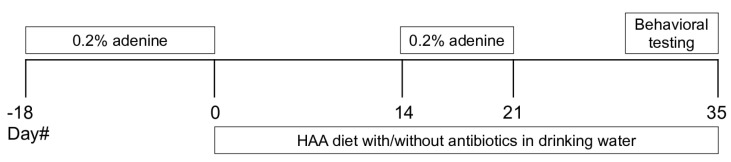
Experimental timeline. Experimental timeline of adenine-induced chronic kidney disease (CKD) in mice, with high-amino-acid (HAA) diet and broad-spectrum antibiotics in drinking water to modulate gut microbiota.

Male and female C57BL/6J mice from Jackson Laboratories (Bar Harbor, ME, USA) aged 10–12 weeks were randomly assigned to experimental groups. For CKD, a nephrotoxic adenine diet was used to induce tubulointerstitial nephritis as previously described [26]. Mice were fed a diet containing 0.2% adenine for 18 days, placed back on regular chow vs. special diet for 2 weeks, and then re-exposed to the adenine diet for 1 week to maintain CKD (Figure 6). Control mice were fed regular chow. Behavior studies and tissue collection were carried out 5–6 weeks after CKD induction and a comparable time in controls. All animal procedures were approved by the Institutional Animal Care & Use Committee at the University of California-Irvine (UCI), under protocol AUP-19-015. The procedures were conducted in accordance with the American Veterinary Medical Association guidelines.

#### 4.1.2. Experimental Diets and Antibiotic Cocktail

Envigo 2020X was used as the base rodent diet (regular chow). CKD induction utilized a 0.2% adenine diet (Dyet# 611732, Dyets Inc., Bethlehem, PA, USA). The high-amino-acid (HAA) diet (Dyet# 611735) was enriched with precursors of indoxyl sulfate, p-cresyl sulfate and TMAO (1.26% tryptophan, 2.88% phenylalanine and 0.6% choline, respectively), providing 3- to 6-fold higher amounts of these dietary nutrients (usual concentrations are 0.2%, 1.0% and 0.12% respectively). For the group of CKD mice assigned to the HAA diet, a 0.2% adenine HAA chow was given in the 1-week adenine re-exposure period (Dyet# 611736, see Figure 6). To suppress intestinal microbiota, subgroups of animals on the HAA diet were treated with broad-spectrum antibiotics in drinking water ×5 weeks following CKD induction (Figure 6); the antibiotic (Abx) cocktail included ampicillin 1 g/L, vancomycin 500 mg/L, neomycin sulfate 1 g/L and metronidazole 1 g/L [51,52]. Thus, we had six experimental groups—CTL, CTL + HAA, CTL + HAA + Abx, CKD, CKD + HAA, CKD + HAA + Abx—to which animals were randomly assigned. Control groups included 8 males and 8 females, while CKD groups included 16 males and 16 females.

#### 4.1.3. Behavior Studies

(1) Spontaneous locomotion was assessed using the Open Field Test [53]. The mouse was placed in an empty white box (12” × 12” with 12” high walls) and movements were tracked for 5 min to measure distance traveled and time in the central zone, and the total distance traveled. Data were analyzed using the SMART 3.0 Open Field Exp Module (Data Sciences International, St. Paul, MN, USA). Total distance traveled in the central zone was used as the primary index of spontaneous locomotion [38,54].

(2) Recognition (working) memory was assessed using the Novel Object Recognition Test [53]. Habituation phase: On days 1–4, the mouse was allowed to explore the empty white box with no objects for 10 min. Familiarization phase: On days 5–6 the mouse was placed in the box with 2 identical objects in specific locations, and allowed to explore for 5 min. On day 7, the mouse was placed back in the testing box with one copy of the object presented previously along with a new, novel object. The discrimination index (DI) was calculated based on the difference in exploration time for the novel vs. the familiar object, dividing this value by the total amount of exploration of both objects. The DI varies between +1 and −1, where a positive score indicates more time spent with the novel object, a negative score indicates more time spent with the familiar object, and a zero score indicates a null preference [55].

#### 4.1.4. Stool 16S rRNA Sequencing

Stool pellets collected from the colon at study termination were stored in 300 μL of RNA/DNA Shield (Zymo Research, Irvine, CA, USA). Samples were extracted at the Zymo Research facility (Irvine, CA, USA), and investigators were blinded to group allocation. Extraction of microbial DNA was performed with a bead-beating step using the ZymoBIOMICS^®^-96 MagBead DNA Kit with an elution volume of 50 μL. Control samples: The ZymoBIOMICS^®^ Microbial Community Standard was used as a positive control for each DNA extraction, and a negative control (i.e., blank extraction control) was included to assess the level of bioburden carried by the wet-lab process. A DNA quality control check was made using Nanodrop^TM^ (Thermo Fisher Scientific, Waltham, WA, USA). The DNA was measured by Qubit and 1.5 ng was used for library construction according to the Illumina 16S Amplicon Library Preparation protocol (Illumina, San Diego, CA, USA) at the UC Irvine Genomics Research and Technology Hub. Libraries were assayed for quality using the Agilent Bioanalyzer 2100 DNA HS chip. The multiplexed library pool with a mock community control sample was sequenced on an Illumina Miseq instrument. Microbiome analysis was carried out at the UCI Microbiome Center. Amplicon sequence variants (ASVs) were inferred in QIIME 2 using DADA2 (qiime dada2 denoise-paired). Demultiplexed paired-end reads were quality filtered, denoised, merged, truncated at 280 (forward) and 202 bp (reverse) based on quality profiles, and screened for chimeras using the consensus method. DADA2 was used to correct sequencing errors and infer exact sequence variants. The resulting ASV table and representative sequences were used for downstream analyses. Taxonomical assignment was achieved using the SILVA database (138.99) trained with the primers used for amplification. Alpha and beta diversity metrics were analyzed within the Vegan package in R (version 2.6-4) [56].

#### 4.1.5. Quantification of Serum Toxins

Serum creatinine was measured using capillary electrophoresis at the O’Brien Kidney Research Core Center (UT Southwestern, Dallas, TX, USA). Cystatin C was measured using the Invitrogen Cystatin C (CST3) Mouse ELISA Kit (EMCST3, Fisher Scientific, Waltham, MA, USA). Gut-derived uremic toxins were quantified using high-performance liquid chromatography–mass spectrometry [57,58]. A 20 μL aliquot of serum was treated with 200 μL of acetonitrile with 0.1% formic acid and internal standards (hydrochlorothiazide 2 μg/mL and salbutamol 5 ng/mL) for protein precipitation. The mixture was vortexed and centrifuged, and the supernatant was evaporated to dryness. The dried extract was reconstituted with 100 uL of 25% acetonitrile. Standards and prepared samples were injected (10 μL) into the HPLC-MS/MS instrument, a Waters Quattro Premier XE equipped with UPLC. For indoxyl sulfate (catalog# I3875, Sigma-Aldrich, St. Louis, MO, USA) and p-cresyl sulfate (p-tolyl sulfate, catalog# P2091, Fisher Scientific), the internal standard used was hydrochlorothiazide (catalog# 5001437615, Fisher Scientific) and analysis was performed in negative ionization mode using multiple reaction monitoring (MRM) MS/MS. For TMAO (catalog# 317594, Sigma), the internal standard used was salbutamol (catalog# S8260, Sigma) and analysis was performed in positive ionization mode [51]. Uremic toxin standard curves were generated in 25% acetonitrile with hydrochlorothiazide 4 μg/mL and salbutamol 10 ng/mL (serial 1/3 dilutions from highest standard 10,000 nM to lowest standard 1.5 nM). The transition (*m*/*z*) values are indoxyl sulfate 211.97 → 80.36, p-cresyl sulfate 186.94 → 107.30, TMAO 76 → 59, hydrochlorothiazide 296.96 → 270.08, salbutamol 240 → 148. CKD serum samples required 20× to 80× dilution to ensure that readouts fell within the standard curve. For human dialysis serum TMAO measurements, samples required 40× dilution.

#### 4.1.6. Brain Microhemorrhage Histology

One brain hemisphere was sectioned into 20 μm coronal sections. Sections were collected every 200 µm and stained with Prussian blue to detect hemosiderin (a marker of iron deposition within microhemorrhages) [59,60,61]. Nuclear fast red was used as a counterstain. Digitized images were analyzed using NIH ImageJ software (version 1.54d) by an observer blinded to the experimental groups, and the number of microhemorrhages was normalized to total brain surface area analyzed per mouse (mm^2^/cm^2^). Average microhemorrhage size was also calculated (mm^2^).

### 4.2. Human Study

The retrospective human study utilized a subgroup of adult hemodialysis patients from the Malnutrition, Diet and Racial Disparities in Chronic Kidney Disease (MADRAD) Study [62,63] (ClinicalTrials.gov NCT01415570) who had biobanked plasma samples under protocol 2012-9045, approved by the Institutional Review Board at UCI. Medical records at the UCI medical center were reviewed to identify participants who had brain MRI scans performed within 2 years of the archived plasma samples. Gut-derived uremic toxins were quantified using high-performance liquid chromatography–mass spectrometry, as described for the mouse samples. Microbleeds were quantified and localized on brain MRI (1.5T or 3T field strength) from 2 mm axial susceptibility-weighted imaging (SWI) [26], by neuroradiologists who were blinded to patient data. Scores were recorded using the Microbleed Anatomical Rating Scale (MARS) [64] and converted into binomial presence or absence of microbleeds for comparison among groups.

### 4.3. Statistical Analysis

Data were analyzed using GraphPad Prism 9 (GraphPad Software, Lo Jolla, CA, USA), SPSS 18.0 software (IMB Corp., Armonk, NY, USA) and SAS version 9.4 (SAS Institute Inc., Cary, NC, USA). For the **mouse study,** normality tests were performed using the D’Agostino–Pearson test. Group comparisons were made using one-way ANOVA. An analysis of sex differences was conducted via two-way ANOVA with post hoc Holm–Sidak test. Associations between variables were determined using Spearman’s correlation coefficient (r) with *p*-values unadjusted for multiple comparisons. Supplemental regression analyses addressed gut-derived toxins adjusted for creatinine only (outcome ~ single toxin + creatinine) and for all toxins in a multivariable model (outcome ~ TMAO + indoxyl sulfate + p-cresyl sulfate + creatinine). Gut microbial alpha- and beta-diversity metrics were analyzed within the Vegan package in R [56]. The Shannon diversity of gut microbiota was analyzed using a linear mixed effects model (nlme package in R, version 3.1-162) after accounting for cage housing (adjustment for animals housed in the same cage). *p* < 0.05 was considered statistically significant. Beta diversity significance was tested using PERMANOVA with adonis as part of the Vegan package in R. To check for dispersion differences, we used beta disper as a part of the Vegan package in R. Observers were blinded to knowledge of the experimental group for behavior studies, stool 16S rRNA sequencing, toxin quantification, and microhemorrhage histology. The mouse study was designed to have 80% power to detect significant differences in microhemorrhage burden in sex-specific analyses. For the **human study**, associations between uremic toxins and microbleeds were examined using logistic regression based on the presence or absence of brain microbleeds. Analyses were adjusted for age, sex, dialysis vintage, and the three uremic toxins of interest (indoxyl sulfate, p-cresyl sulfate, TMAO). Mouse and human data are presented as mean ± SEM.

### 4.4. Data Availability

All raw data from this study are available in Dryad datasets (https://10.5061/dryad.hx3ffbgr4, accessed on 29 June 2026). All microbial sequencing data generated in this study have been deposited in the NCBI Sequence Read Archive under BioProject ID PRJNA954040. A complete list of individual accession numbers is provided in the Dryad link.

## 5. Conclusions

Our study confirms that the gut microbiota is the primary source of circulating p-cresyl sulfate, indoxyl sulfate and TMAO in CKD animals and that antibiotic treatment markedly suppresses these toxins. These toxins correlated with brain microhemorrhage burden and neurobehavior changes (Figure 5). Sex differences were observed; in male animals, higher TMAO was associated with increased microhemorrhages (this was also observed in the human study of hemodialysis patients), whereas in female mice, pCS was associated with microhemorrhage burden (Figure 4). Intriguingly, the suppression of toxins with antibiotics improved working memory in male animals. Correlation analyses suggest that the gut-derived uremic toxins may influence brain health via microhemorrhage-dependent and -independent pathways. Notwithstanding complexities of the kidney–gut–brain axis, the impact of CKD on cerebral microhemorrhage development remains a consistent finding across multiple studies.

## Figures and Tables

**Table 1:** Parameters (mean ± standard error) at study termination in control (CTL) and chronic kidney disease (CKD) animals; combined female and male animal data. Toxin levels were similar across the three CTL groups. Abx = antibiotics in drinking water; CMH = cerebral microhemorrhages; HAA = high-amino-acid diet. Data presented as mean ± standard error; ANOVA with post hoc Holm–Sidak test; * *p* < 0.05 compared with CTL; ^#^ *p* < 0.05 comparing CKD + HAA or CKD + HAA + Abx with untreated CKD.

	CTL *n* = 16	CTL + HAA *n* = 16	CTL + HAA + Abx *n* = 16	CKD *n* = 32	CKD + HAA *n* = 32	CKD + HAA + Abx *n* = 32
Creatinine (mg/dL)	0.06 ± 0.004	0.06 ± 0.003	0.06 ± 0.003	0.19 ± 0.012 *	0.13 ± 0.007 *^#^	0.12 ± 0.006 *^#^
Cystatin C (mg/L)	0.34 ± 0.05	0.21 ± 0.04	0.18 ± 0.03	1.85 ± 0.26 *	1.01 ± 0.12 ^#^	0.98 ± 0.12 ^#^
p-Cresyl sulfate (µM)	1.8 ± 0.6	1.6 ± 0.6	Not detected	14.2 ± 3.6 *	16.7 ± 3.7 *	Not detected ^#^
Indoxyl sulfate (µM)	1.8 ± 0.2	1.7 ± 0.2	Not detected	11.0 ± 1.6 *	5.8 ± 0.8 *^#^	Not detected ^#^
TMAO (µM)	2.4 ± 0.8	41.7 ± 17.2	0.2 ± 0.02	12.7 ± 3.5	66.5 ± 12.9 *^#^	0.2 ± 0.02
Shannon diversity index	4.3 ± 0.1	4.1 ± 0.1	3.4 ± 0.5	4.2 ± 0.1	4.1 ± 0.1	4.0 ± 0.2
CMH count per cm^2^	0.68 ± 0.10	0.66 ± 0.15	1.11 ± 0.21	1.70 ± 0.24 *	1.32 ± 0.17	1.15 ± 0.15

**Table 2:** Behavior testing in control (CTL) and chronic kidney disease (CKD) animals; combined male and female animal data. Abx = antibiotics in drinking water; HAA = high-amino-acid diet. Data presented as mean ± standard error; ANOVA with post hoc Holm–Sidak test; * *p* < 0.05 compared with CTL; ^#^ *p* < 0.05 compared with CKD.

	CTL *n* = 16	CTL + HAA *n* = 16	CTL + HAA + Abx *n* = 16	CKD *n* = 32	CKD + HAA *n* = 32	CKD + HAA + Abx *n* = 32
Distance traveled in central zone (cm)	604 ± 60 ^#^	558 ± 57 ^#^	482 ± 44 ^#^	279 ± 30 *	380 ± 35 *	283 ± 32 *
Discrimination index	0.26 ± 0.06	0.25 ± 0.07	0.25 ± 0.07	0.18 ± 0.05	0.15 ± 0.05	0.28 ± 0.06

**Table 3:** Clinical characteristics, uremic toxin levels and microbleed presence in a cohort of chronic hemodialysis patients (16 of 30 patients had microbleeds, including 4 patients with microbleeds in >1 brain region). Data shown as mean ± SEM or percentage of cohort.

Hemodialysis Patients		*n* = 30
Males (%)		50%
Age (years)		58.5 ± 3.1
Dialysis vintage (months)		54.1 ± 3.1
Antiplatelet or anticoagulation medication use		10/30 (33%)
Comorbid conditions	Hypertension	27/30 (90%)
	Diabetes mellitus	14/30 (47%)
	Stroke	7/30 (23%)
	Congestive heart failure	10/30 (33%)
Uremic toxins (µM)	p-Cresyl sulfate	43.9 ± 7.4
	Indoxyl sulfate	9.5 ± 0.9
	Trimethylamine N-oxide	5.5 ± 0.5
Brain microbleeds	Infratentorial	3 (10%)
	Deep	6 (20%)
	Lobar	11 (37%)

## Data Availability

The data presented in this study are openly available in Dryad datasets at https://doi.org/10.5061/dryad.hx3ffbgr4, accessed on 29 June 2026.

## Supplementary Materials

The following supporting information can be downloaded at: https://www.mdpi.com/article/10.3390/ijms27136020/s1.
