# Supplementary material for: Investigating the Kidney–Gut–Brain Axis in CKD: Uremic Toxins and Brain Microhemorrhages
Source: Int J Mol Sci. 2026 Jul 4;27(13):6020. doi: 10.3390/ijms27136020 (PMC13361717; doi:10.3390/ijms27136020)
Supplement: Supplementary file 1 [file ijms-27-06020-s001.zip › ijms-4267961-supplementary.pdf]

Supplemental Table S1. Study parameters in control (CTL) and chronic kidney disease (CKD) animals, female vs male mice. Abx = antibiotics in drinking water; CMH = cerebral microhemorrhages; HAA = high amino acids diet; TMAO = trimethylamine N-oxide. Data presented as mean ± standard error; * $P < 0.05$ compared to CTL of same sex; $^{\#}$ $P < 0.05$ compared to CKD of same sex; † $P < 0.05$ for sex differences within same experimental group.

	Male						Female					
	**CTL** n=8	**CTL+HAA** n=8	**CTL+HAA+Abx** n=8	**CKD** n=16	**CKD+HAA** n=16	**CKD+HAA+Abx** n=16	**CTL** n=8	**CTL+HAA** n=8	**CTL+HAA+Abx** n=8	**CKD** n=16	**CKD+HAA** n=16	**CKD+HAA+Abx** n=16
**Body weight (g)**	30±0.6$^{\#\dagger}$	28±0.8$^{\#\dagger}$	28±0.5$^{\#\dagger}$	23±0.3*†	23±0.5*†	23±0.4*†	23±0.5$^{\#\dagger}$	22±0.6†	23±0.5$^{\#\dagger}$	21±0.3*†	21±0.4†	22±0.3†
**Creatinine (mg/dL)**	0.05±0.002$^{\#\dagger}$	0.06±0.005$^{\#}$	0.05±0.002$^{\#}$	0.24±0.01*†	0.16±0.003$^{*\#\dagger}$	0.14±0.008$^{*\#\dagger}$	0.08±0.004$^{\#\dagger}$	0.07±0.004$^{\#}$	0.06±0.005$^{\#}$	0.14±0.009*†	0.10±0.009$^{\#\dagger}$	0.09±0.005$^{\#\dagger}$
**Cystatin C (mg/L)**	0.37±0.11$^{\#}$	0.09±0.06$^{\#}$	0.07±0.03$^{\#}$	2.98±0.32*	1.38±0.18$^{*\#}$	1.34±0.19$^{\#}$	0.31±0.02$^{\#}$	0.29±0.03$^{\#}$	0.28±0.03$^{\#}$	0.72±0.07*	0.62±0.07*	0.60±0.06*
**p-Cresyl sulfate (µM)**	2.3±1.0$^{\#}$	3.0±1.0$^{\#\dagger}$	Undetectable$^{\#}$	27.4±5.5*†	32.5±4.8*†	Undetectable #	0.4±0.1	0.2±0.02$^{\#\dagger}$	Undetectable #	0.8±0.1†	0.7±0.1†	Undetectable $^{\#}$
**Indoxyl sulfate (µM)**	1.5±0.2$^{\#}$	1.7±0.2$^{\#}$	Undetectable$^{\#}$	17.3±1.9*†	8.9±1.1$^{*\#\dagger}$	Undetectable #	2.1±0.4$^{\#}$	1.7±0.3$^{\#}$	Undetectable *#	4.3±0.5*†	2.7±0.3$^{\#\dagger}$	Undetectable *#
**TMAO (µM)**	0.09±0.02 †	0.29±0.07†	0.15±0.04	1.76±0.37*†	5.52±1.07$^{*\#\dagger}$	0.08±0.003$^{\#\dagger}$	4.8±1.2†	78.0±26.6$^{*\#\dagger}$	0.2±0.02	20.0±4.2†	119.9±13.9$^{*\#\dagger}$	0.3±0.02†
**Shannon diversity index**	3.825±0.1†	3.709±0.1†	3.004±0.8	3.887±0.1†	3.629±0.1†	3.466±0.4	4.806±0.1†	4.530±0.1*†	3.890±0.6	4.595±0.1†	4.498±0.1*†	4.439±0.2
**CMH per cm^2**	0.54±0.10	0.83±0.22	1.19±0.33	1.35±0.27	1.03±0.19	0.75±0.16	0.81±0.17	0.48±0.20$^{\#}$	1.04±0.28	2.04±0.38*	1.60±0.27	1.55±0.23*
**Total CMH area (mm^2/cm^2)**	0.0006±0.0002	0.0008±0.0003	0.0007±0.0002	0.0008±0.0002	0.0006±0.0002	0.0005±0.0001	0.0008±0.0002	0.0003±0.0001$^{\#}$	0.0014±0.0005	0.0019±0.0004	0.0010±0.0002	0.0010±0.0002
**Average CMH size (mm^2)**	0.0005±0.0001	0.0008±0.0002	0.0005±0.0001	0.0008±0.0001	0.0007±0.0001	0.0006±0.0001	0.0010±0.0003	0.0003±0.0002$^{\#}$	0.0011±0.0004	0.0009±0.0001	0.0006±0.0001	0.0007±0.0001
**Distance traveled in central zone (cm)**	460±69$^{\#\dagger}$	360±38$^{\#\dagger}$	382±40$^{\#\dagger}$	150±22*†	229±26*†	170±29*†	749±68$^{\#}$	756±33$^{\#}$	582±62$^{\#}$	399±29*	531±37*	434±29*
**Total distance traveled (cm)**	1649±195†	1504±124†	1551±186	934±111*†	1149±54$^{*\#\dagger}$	876±91*†	2580±163	2409±102	2198±206	2025±78*	2142±82*	1971±116*
**Discrimination index**	0.43±0.05†	0.38±0.04	0.36±0.08	0.24±0.06	0.22±0.07	0.45±0.07$^{\#\dagger}$	0.26±0.04	0.31±0.07	0.22±0.10	0.26±0.05	0.27±0.05	0.27±0.06

Supplemental Table S2. Correlation analyses with serum levels of uremic toxins in mouse control and CKD groups. Only significant correlations are shown. Data are Spearman's correlation coefficient (r); *P < 0.05, **P < 0.01, ***P < 0.001, ****P < 0.0001.

	Cerebral microhemorrhages per cm^2	Distance traveled in central zone, cm (Open Field Test)	Discrimination index (Novel Object Recognition test)
Overall mouse cohort			
**Creatinine (mg/dL)**		-0.577****	
**p-Cresyl sulfate (µM)**		-0.233**	
**Indoxyl sulfate (µM)**		-0.232**	
**Trimethylamine N-oxide (µM)**	0.278**	0.300***	-0.295***
Males			
**Creatinine (mg/dL)**		-0.66****	-0.26*
**p-Cresyl sulfate (µM)**			-0.31*
**Indoxyl sulfate (µM)**			-0.26*
**Trimethylamine N-oxide (µM)**	0.30*	-0.28*	
Females			
**Creatinine (mg/dL)**	0.28*	-0.49****	
**p-Cresyl sulfate (µM)**	0.27*		
**Indoxyl sulfate (µM)**			
**Trimethylamine N-oxide (µM)**			

Overall mouse cohort: Creatinine and gut-derived toxins			
	p-Cresyl sulfate (µM)	Indoxyl sulfate (µM)	Trimethylamine N-oxide (µM)
**Creatinine (mg/dL)**	0.456****	0.560****	0.252**

Supplemental Table S3. Univariable and multivariable regression analysis of uremic toxins and cerebral microhemorrhage burden (CMH counts per cm^2) in the overall mouse cohort, and by sex. Single-toxin model refers to each gut-derived toxin adjusted for creatinine only (outcome ~ one toxin + creatinine). Multi-toxin adjusted model refers to multivariable adjustment with indoxyl sulfate, p-cresyl sulfate and TMAO combined with creatinine (outcome ~ TMAO + IS + pCS + creatinine). β: regression coefficient; CI: confidence interval.

Cohort	Variable	Univariable β (95% CI)	P value of univariable model	Single-toxin adjusted model β (95% CI)	P value of toxin single-toxin adjusted model	Multi-toxin adjusted model β (95% CI)	P value of multi-toxin adjusted model
OVERALL COHORT	TMAO	0.0051 (0.0016, 0.0086)	<0.01	0.0054 (0.0019, 0.0088)	<0.01	0.0061 (0.0025, 0.0096)	<0.01
	Indoxyl sulfate	0.0165 (-0.0109, 0.0438)	0.24	-0.0013 (-0.0407, 0.0380)	0.95	-0.0202 (-0.0698, 0.0295)	0.42
	P-Cresyl sulfate	0.0064 (-0.0043, 0.0171)	0.24	0.0011 (-0.0117, 0.0139)	0.87	0.0060 (-0.0098, 0.0218)	0.45
	Creatinine	2.2850 (-0.3299, 4.8990)	0.09	---	---	3.9630 (0.0616, 7.8650)	<0.05
MALES	TMAO	0.0608 (-0.0060, 0.1276)	0.07	0.0433 (-0.0252, 0.1118)	0.21	0.0104 (-0.0667, 0.0875)	0.79
	Indoxyl sulfate	0.0262 (0.0030, 0.0495)	<0.05	0.0239 (-0.0103, 0.0581)	0.17	-0.0089 (-0.0504, 0.0325)	0.67
	P-Cresyl sulfate	0.2297 (-0.0098, 0.4692)	0.06	0.0134 (0.0027, 0.0242)	<0.05	0.0133 (-0.0008, 0.0274)	0.06
	Creatinine	2.1000 (-0.3872, 4.5880)	0.10	---	---	1.5530 (-2.0560, 5.1620)	0.39
FEMALES	TMAO	0.0035 (-0.0010, 0.0080)	0.37	0.0025 (-0.0017, 0.0068)	0.24	0.0030 (-0.0016, 0.0076)	0.19
	Indoxyl sulfate	0.1285 (-0.0087, 0.2658)	0.61	0.0310 (-0.1307, 0.1927)	0.70	-0.0827 (-0.2884, 0.1230)	0.42
	P-Cresyl sulfate	0.2297 (-0.0098, 0.4692)	0.06	0.1151 (-0.1221, 0.3522)	0.34	0.1351 (-0.1387, 0.4089)	0.33
	Creatinine	9.4570 (2.9830, 15.9300)	<0.01	---	---	13.0000 (3.7900, 22.2100)	<0.01
